# Cesarean hysterectomy in a hybrid operating room for placenta percreta: a report of three cases

**DOI:** 10.1186/s40981-019-0230-5

**Published:** 2019-02-12

**Authors:** Takashige Yamada, Eriko Hirahata, Naho Ihara, Daisuke Nishimura, Kei Inoue, Jungo Kato, Hiromasa Nagata, Shizuka Minamishima, Hiroshi Morisaki

**Affiliations:** Department of Anesthesiology, Keio University School of Medicine, 35 Shinanomachi, Shinjuku, Tokyo, 160-8582 Japan

**Keywords:** Placenta percreta, Hybrid operating room, Endovascular arterial embolization, Cesarean hysterectomy

## Abstract

**Background:**

Placenta percreta is the most severe abnormality in invasive placenta and often treated with cesarean hysterectomy. Endovascular embolization for placental abnormality is known to reduce bleeding from the placental bed and from the abnormal neovasculature surrounding the uterus. We describe three cases of placenta percreta treated with uninterrupted cesarean hysterectomy and embolization performed using a hybrid operating room (HOR).

**Case description:**

Cases were two placenta previa percretas and an impending uterine rupture with placenta percreta, treated with elective cesarean hysterectomy in HOR. Planned conversion of spinal to general anesthesia was performed after the fetal delivery. Immediate embolic devascularization of abnormal neovasculature was directly observed and facilitated adhesiolysis. Surgical blood losses were 1850 g, 2500 g, and 1180 g, respectively.

**Conclusion:**

Cesarean hysterectomy combined with endovascular embolization in the HOR for placenta percreta is an advantageous option to enhance patient safety by multidisciplinary approach without patient transfer.

## Background

Placenta percreta is the most severe abnormality in invasive placenta, resulting in life-threatening hemorrhage during delivery. The mortality rate is reported up to 7% [1], and the mean blood loss exceeds 12,000 g [2]. Among several strategies to manage such high-risk placental abnormalities, cesarean hysterectomy combined with radiological intervention has been recognized to play a crucial role to reduce postpartum massive hemorrhage [3]. We believe that the HOR is particularly useful for patients with placenta percreta who undergo both arterial embolization and cesarean hysterectomy because the use of HOR precludes patient transfer and enables to minimize the time loss and direct inspection of the effects of embolization. Clark et al. reported successful management of two cases with placenta percreta by cesarean section and uterine artery embolization in the HOR [4]. One of them underwent subsequent hysterectomy. However, detailed information was unavailable. In particular, the direct observation during embolization was not recorded also in other case reports to date [5, 6]. We describe three cases of patients who underwent such combined therapy in the HOR.

## Case presentation

### Case 1

A 40-year-old woman, with two previous cesarean sections, presented with antepartum hemorrhage due to complete placenta previa at 30 weeks of gestation. Magnetic resonance imaging (MRI) suggested the presence of placenta percreta. An elective cesarean hysterectomy was planned at 32 weeks and 2 days of gestation under combined spinal and epidural anesthesia (CSEA). Two 18-gauge peripheral venous catheters, central venous catheter via jugular vein, and arterial catheter in radial artery were placed before surgery. After cesarean delivery of the fetus under supine position, the uterus was closed, leaving the placenta in situ. Interventional radiologists inserted 5-French arterial sheaths via the femoral artery, and CSEA was converted to general endotracheal anesthesia with low concentration of desflurane and fentanyl. Bilateral uterine arteries were embolized with *n*-butyl-2-cyanoacrylate, and sufficient flow reduction was confirmed with both angiogram and direct observation. The estimated blood loss was 1850 g during the procedure, and 400 ml of autologous blood was transfused. She was discharged uneventfully.

### Case 2

A 34-year-old woman, with a history of previous cesarean section, presented with preterm labor at 28 weeks and 6 days of gestation. Ultrasonography and MRI revealed placenta accreta on a thinning uterine wall along the previous scar and suggested bloody ascites. Intensive tocolysis failed to prevent increasing hemoperitoneum, and an elective cesarean hysterectomy was performed under CSEA for impending uterine rupture at 29 weeks and 1 day of gestation in the HOR. Two 18-gauge peripheral venous catheters and arterial catheter in radial artery were placed before surgery. The hemoperitoneum weighed 400 g was observed after laparotomy, and the fetus was safely delivered. The uterus was closed immediately, leaving the placenta in situ. After the insertion of 5-French arterial sheaths via the femoral artery, CSEA was converted to low-dose sevoflurane-remifentanil anesthesia with endotracheal intubation, and the interventional radiologists embolized bilateral internal iliac arteries and left uterine artery with gelatin sponge. The embolization stopped bleeding from the protruded placenta. Hysterectomy was accomplished with 1180 g of blood loss, and 2 units of packed red blood cells and 1 unit of fresh frozen plasma were transfused. She was discharged uneventfully.

### Case 3

A 35-year-old woman, with a history of several laparoscopic gynecological surgeries, presented with preterm labor. MRI revealed placenta protruding toward the rectum and bilateral internal iliac vessels, suggesting placenta percreta and marginal placenta previa. Cesarean hysterectomy combined with endovascular treatment was planned at 35 weeks and 4 days of gestation in the HOR. Twenty-gauge and 18-gauge peripheral venous catheters in the forearm and arterial catheter in the radial artery were placed before surgery. Cesarean section was performed following ureteral stents placement under CSEA. After delivery of the fetus, the uterus was closed leaving the placenta in situ. General anesthesia was induced after bilateral 5-French femoral arterial sheaths were placed. Bilateral uterine arteries were embolized with gelatin sponge under low-dose desflurane-fentanyl anesthesia with endotracheal intubation. A direct visualization of embolic devascularization in surgical view could not be obtained because the placenta percreta was located on the posterior wall. Collaborative hysterectomy including partial cystectomy and prudent adhesiolysis took 340 min, with 2500 g of blood loss and 1200 ml of autologous blood transfusion. She was discharged uneventfully.

## Discussion

This is the first case series of cesarean hysterectomy combined with on-site endovascular arterial embolization for placenta percreta, the most severe placental abnormality. Although standard treatment has not yet been established, endovascular balloon occlusion or embolization is frequently used to control bleeding [7]. In our series, cesarean section was performed in supine position under CSEA. After cesarean delivery, the uterus was closed without any placental manipulation. Subsequently, general endotracheal anesthesia was induced, followed by arterial embolization and hysterectomy. Such technique offers advantages of CSEA during cesarean section and advantages of general anesthesia during embolization procedure and hysterectomy. This approach is ideal for unstable parturients who cannot afford transfer between the operating theater and interventional radiology suite. Additionally, in HOR, the effectiveness of the treatment can be evaluated with direct visualization. A certain control of the blood flow to the uterus via surrounding abnormal neovasculature is a key to reduce bleeding during hysterectomy. In our hospital, confirmed placenta percreta, increta, and accreta are always treated in HOR, and suspected accreta and placenta previa are encouraged to be treated in HOR.

The management strategy for high-risk obstetric procedures includes scheduled delivery, multidisciplinary care, and surgical planning, which is classified into three kinds of surgery: cesarean hysterectomy, cesarean delivery while leaving the placenta in situ, and cesarean delivery followed by focal resection of the adherent placenta [8, 9]. Because the manual attempt to remove adhered placenta leads to critical bleeding, hysterectomy or leaving placenta strategies are commonly advocated.

Alternatively, uterine conservative management combined with endovascular embolization may be considered [10]. Indeed, we initially planned for preservation management if case 3 has extreme adhesion or invasion, but was not performed eventually. Conservative management might involve serious complications, such as post-partum hemorrhage and infection, which could require second embolization or unplanned hysterectomy. These serious complications were reported in 56% of patients on conservative management, including 22% of emergency hysterectomy cases in a recent literature [10].

Angstman et al. reported staged hysterectomy combined with uterine arterial embolization for placenta accreta, involving transportation to and from radiology suite before hysterectomy after cesarean delivery [11]. Their scheme significantly reduced blood loss compared with primary cesarean hysterectomy, whereas 3 of 11 women cannot be transported to the radiological room because of placental dehiscence and subsequent massive hemorrhage immediately after cesarean section. Besides, the high incidence of adverse events during in-hospital transportation of unstable patients has been appreciated [12], and longer transport time is associated with larger blood loss. The advantages of immediateness to embolization are underscored as cesarean section in interventional radiology room was previously attempted [5]. Although our patients were all hemodynamically stable before embolization, HOR utilization could provide more benefits in case of emergency due to hemorrhage or deteriorating fetal conditions.

A systematic review recently indicated that endovascular interventions for abnormal placentation reduced the total blood loss by 893 g on average [3]. Owing to no control data of blood loss in this case series, we may not be allowed to make any conclusive comments on this issue. Among deliveries complicated by placenta percreta reported previously [2], however, we believe that the clinical course of 1200 to 2500 g blood loss in all three patients and their subsequent uneventful discharge could be considerably affordable.

Konishi et al. reported the case of intraoperative embolization in the HOR for placenta percreta, in which they used a high concentration of inhaled anesthetics for the purpose of uterine relaxation with the intention to prevent placental dehiscence after delivery [6]. We did not use myometrial relaxants because prolonged relaxation potentially leads an atonic hemorrhage. Constriction of tortuous vessels by oblique muscle contraction is a major mechanism for hemostasis for most placental site, except for an abnormally invading area, and a uterotonic drug is positively used in the conservative management of leaving the placenta in situ [13]. We believe that intensive relaxation is not absolutely necessary and hemorrhage will be controllable when the uterine incision line was enough apart from the placental edge and immediate embolization was ready.

Several issues should be considered to perform cesarean hysterectomy with endovascular embolization in the HOR. First, in addition to the presence of interventional radiologists and surgeons for the pelvic region, the installation of HOR might be limited even in hospitals where high-risk obstetric procedures are provided. Such high-risk parturients complicated by abnormal placenta should be transferred in advance to such institution. Second, in case of emergency cesarean section for high-risk parturients, the HOR might not be always available due to other ongoing surgeries. Third, some operating tables in the HOR cannot rapidly transform into the lithotomy position in cases where transvaginal treatment is required. In our cases, all parturients underwent cesarean delivery in the supine position to start endovascular embolization as soon as possible.

We describe three cases of successful cesarean hysterectomy for placenta percreta. Using the HOR may be advantageous to patients requiring arterial embolization between cesarean section and hysterectomy to prevent massive hemorrhage. The direct visualization during embolization could serve as a helpful reference for effective embolization.

## Abbreviations

CSEA: Combined spinal and epidural anesthesia
HOR: Hybrid operating room
MRI: Magnetic resonance imaging
